# Development and Application of Rapid Clinical Visualization Molecular Diagnostic Technology for *Cryptococcus neoformans*/*C. gattii* Based on Recombinase Polymerase Amplification Combined With a Lateral Flow Strip

**DOI:** 10.3389/fcimb.2021.803798

**Published:** 2022-01-12

**Authors:** Lei Wang, Yan Wang, Fang Wang, Mengdi Zhao, Xuzhu Gao, Huimin Chen, Na Li, Qing Zhu, Lipin Liu, Wenjun Zhu, Xia Liu, Yujiao Chen, Ping Zhou, Yingzhi Lu, Kun Wang, Weiguo Zhao, Wei Liang

**Affiliations:** ^1^ Department of Central Laboratory, Lianyungang Hospital Affiliated to Jiangsu University, Lianyungang, China; ^2^ School of Biotechnology, Jiangsu University of Science and Technology, Zhenjiang, China; ^3^ Department of Materials Science and Engineering, Suzhou University of Science and Technology, Suzhou, China; ^4^ Lianyungang Second People’s Hospital Affiliated to Bengbu Medical College, Lianyungang, China; ^5^ Lianyungang Hospital Affiliated to Xuzhou Medical University, Lianyungang, China

**Keywords:** *C. neoformans*, *C. gattii*, CAP64, base mismatches, RPA-LFS

## Abstract

*Cryptococcus neoformans* (*C. neoformans*)/*C. gattii* can easily invade the human central nervous system and cause cryptococcal meningitis (CM). The clinical fatality rate of these fungi is extremely high and causes more than 180,000 deaths worldwide every year. At present, the common clinical identification methods of these fungi are traditional culture methods and Indian ink staining. In addition, enzyme-linked immunosorbent assay (ELISAs), polymerase chain reaction (PCR), real-time quantitative PCR detecting system (qPCR), mass spectrometry, and metagenomic next-generation sequencing (mNGS) have also been applied to detect these fungus. Due to the rapid progress of meningitis caused by *C. neoformans*/*C. gattii* infection, there is a desperate need for fast, sensitive, and on-site detection methods to meet the clinical diagnosis. Recombinase polymerase amplification (RPA) is a promising isothermal amplification technique that can compensate for the shortcomings of the above techniques, featuring short reaction time, high specificity, and high sensitivity, thus meeting the demand for in-field detection of *C.neoformans*/*C. gattii*. In our study, RPA- lateral flow strip (LFS) was used to amplify the capsule-associated gene, *CAP64*, of *C. neoformans*/*C. gattii*, and the primer-probe design was optimized by introducing base mismatches to obtain a specific and sensitive primer-probe combination for clinical testing, and specificity of the detection system was determined for 26 common clinical pathogens. This system was developed to obtain results in 20 min at an isothermal temperature of 37°C with a lower limit of detection as low as 10 CFU/μL or 1 fg/μL. A total of 487 clinical samples collected from multicenter multiplexes were tested to evaluate the detection performance of the RPA-LFS system, which revealed that the system could specifically detect *C. neoformans*/*C. gattii*, meeting the need for rapid, specific, and sensitive detection.

## Highlights

The RPA-LFS established in this study can detect *C*. *neoformans/C*. *gattii* at 37°C for 20 min, which allows for rapid and accurate diagnosis of critically ill patients. The traditional culture method can delay the diagnosis of CM and cause irreversible damage due to its time-consuming nature. With the method established in this study, test results are provided rapidly, and through the correct and timely administration of drugs and aggressive treatment by clinicians, the maximum number of critically ill patients can be saved.

## Introduction

*Cryptococcus neoformans* and *Cryptococcus gattii* are the two main pathogenic cryptococci that cause human infections (Torda et al., 2001). CM is fungal meningitis caused by *C. neoformans* and *C. gattii* infection. Untreated CM has a 100% mortality rate, a 10–40% mortality rate after treatment, and a 20–25% recurrence rate in survivors. Thus, the clinical mortality rate of these fungi is extremely high, causing more than 180,000 deaths worldwide each year. Early diagnosis is important for treatment of this disease (Shaheen et al., 2018). *C*. *neoformans* is the main pathogen of cryptococcosis and mainly affects immunocompromised individuals (Rodrigues et al., 1999; Voelz and May 2010), showing a preference for CNS infection (Rodrigues et al., 2007; Bielska and May 2016; May et al., 2016). In immunocompetent individuals, *C*. *gattii* can establish Cryptococcus infections and infect the lungs, exhibiting a high pathogenicity that is difficult to treat. *C*. *neoformans* is a more common causative agent of cryptococcal infections in China and elsewhere compared to *C. gattii* (Bromilow and Corcoran, 2007; Chen et al., 2008). *C. neoformans* and *C. gattii* are yeasts of the genus Cryptococcus, round or ovoid, budding, with a fungal body size of 4–20 μm, and surrounded by a thick capsule of polysaccharide. The major pathogenic serotypes include serotypes A, B, C, D, and AD; most of the clinical isolates are serotypes A and D (Huang et al., 2018). The susceptible population is pigeon breeders, patients with chronic debilitating diseases and immune system disorders, such as diabetes, renal failure, liver cirrhosis, malignant lymphoma, leukemia, AIDS, organ transplantation, and patients with long-term use of high-dose glucocorticoid therapy and other immunosuppressive agents. The clinical presentation is chronic or subacute onset, with progressive worsening of symptoms that include swelling of the brain and headache, which may be accompanied by fever, nausea, vomiting, and irritability (George et al., 2017; Rajasingham et al., 2017).

Traditional culturing methods and Indian ink staining are used to identify *C. neoformans/C. gattii* organisms to confirm diagnosis of this disease. The method of traditional culturing methods is easy to delay the disease due to the long detection period (Cano et al., 2020). The main disadvantages of Indian ink staining are the low sensitivity, the tendency of the staining solution to dry, and the tendency of the stained sediment to deposit on the cells and affect the reading of the smear; this method has a high false-negative rate and cannot be used as a reliable basis for clinical diagnosis. Gressler et al. (2021) used an indirect enzyme-linked immunosorbent assay (ELISA) to detect anti-*C. neoformans* IgG in the blood of patients with cryptococcal meningitis with a specificity of 96.7%, but this immunology-based method suffers from poor reproducibility and interference with autoantibodies and heterophilic antibodies is prone to false positives. Regardless of instrumentation and manual operation, there are many interfering factors, the most influential of which are temperature and time. Therefore, the use of this method is limited in clinical practice. Rappelli (1998) reported a nested PCR-based assay for the detection of *C*. *neoformans* in cerebrospinal fluid; Qishui et al. (2012) established a TaqMan qPCR method for quantitative detection of *C. neoformans* genomic DNA using ITS-rDNA sequences as the detection target; these molecular diagnostic methods based on nucleic acid amplification rely on expensive thermal cycling instruments and specially trained laboratory personnel and sites, which cannot meet the needs of field detection. Mass spectrometry usually requires coupling with liquid or gas chromatography, and the instruments require careful maintenance and suffer from poor qualitative capabilities, making them difficult to apply on a large scale in primary care settings. mNGS was used by Xing et al. (2019) to sequence and analyze samples from 12 consecutive non-HIV-infected chronic or chronic HIV/AIDS patients and was compared to ELISA and Indian ink negative staining methods; the detection rate was only 81.82% and the cost of the assay was high and the testing period was considered long.

Thus, there is a need for an assay that can combine the advantages of the above methods and address the shortcomings to meet the demand for rapid, sensitive, and portable rapid diagnosis. One possibility is recombinase polymerase amplification (RPA), a promising new isothermal amplification technology. Lateral flow strip (LFS) can be coupled with RPA technology to detect various nucleic acid substances. Currently, RPA-LFS is used to test various pathogenic microorganisms, including *L*. *monocytogenes* (Wang et al., 2019) and *Vibrio alginolyticus* (Dong et al., 2019), viruses such as small ruminant distemper virus (Yang et al., 2017) and canine microvirus (Liu et al., 2018), and *Mycoplasma pneumoniae* (Liu et al., 2019a). In this study, we propose to establish a rapid detection technique for *C. neoformans/C. gattii* using RPA-LFS.

## Materials And Methods

### Ethics Statement

This study was approved by the Medical Ethics Committee of the Second People’s Hospital of Lianyungang City (Permit Number: 2020013). The clinical strains were collected from 2020 to 2021 and isolated from cerebrospinal fluid. All samples were obtained with written consent of every patient.

### Clinical Specimens and Strains

*C. neoformans* (ATCC No. 14116/204092/32045/34877/66031/76848) and *C. gattii (*ATCC No. 34877*)* were purchased from Shanghai Covey Chemical Technology Co., Ltd. *Acinetobacter baumannii*, *Candida albicans*, *Enterobacter cloacae*, *Enterococcus faecium*, *Escherichia coli* O157, *Mycobacterium tuberculosis* H37Ra, *Pseudomonas aeruginosa*, *Staphylococcus aureus*, *Staphylococcus capitis*, *Staphylococcus epidermidis*, *Staphylococcus haemolyticus*, *Staphylococcus hominis*, *Staphylococcus saprophyticus*, *Staphylococcus warneri*, *Stenotrophomonas maltophilia*, *Streptococcus pneumonia*, *Viridans streptococci*, *Klebsiella pneumoniae*, *Haemophilus influenzae*, *Listeria monocytogenes*, *Neisseria meningitidis*, *Pseudostelium portuguensis*, *Pseudostelium tropicalum*, *Pseudomonas graminearum*, *Pseudostelium glossyum*, *Pseudomonas dublinensis*, and 26 other bacterial species were provided by our laboratory (**Supplementary Table S1**), and 487 cerebrospinal fluid samples from patients with suspected CM were collected from various hospitals in Lianyungang and hospitals in surrounding cities, including Lianyungang First People’s Hospital, Lianyungang Second People’s Hospital, Lianyungang Third People’s Hospital, Huai’an First People’s Hospital, and Suqian People’s Hospital.

### DNA Extraction

All bacterial strains were incubated at 100°C for 10 min before serving as templates. If not specified, 1 µL of the heat-treated culture at 10^5^ CFU/mL was used as the template. For *C. neoformans* and other fungi, genomic DNA was extracted and purified using a GeneJET Genomic DNA Purification Kit (Tiangen Biotech Co., Ltd., Beijing, China) from the culture or cerebrospinal fluid sample as per the manufacturer’s instruction. The extracted genomic DNA was quantified using a Qubit 4 fluorometer (Thermo Fisher Scientific) as per the manufacturer’s instruction.

### Primers and Probes

RPA primers were designed with Primer Premier 5.0 software (Premier Biosoft International, CA, USA) according to the sequences of the capsular-related genes *CAP10*, *CAP59*, and *CAP64* from the *C. neoformans/C. gattii* genome (GenBank: AE017341.1). For the primers, after the sequence of a particular targeting region were inputted, the parameters were set as follows: The product size was set as min. 100 and max. 300. The primer size was set as min. 30 and max. 35, and a good-performing forward primer extending approximately 15 bp from the 3′ ends was selected as a probe. The possibility of pairing between the forward and reverse primers and the probe was manually checked. Primers with sequence pairing of more than three consecutive bases (and more than one base if at the 3′ end) were abandoned. The sequences of the primers and probes were then confirmed for species specificity using Primer-BLAST on the NCBI website (https://www.ncbi.nlm.nih.gov/tools/primer-blast).

### RPA Procedure and Electrophoresis

RPA is a multi-enzyme system that consists of recombinase uvsX, polymerase Bsu, and single-stranded binding protein gp32. Bsu binds the primer and initiates nucleic acid amplification (**Supplementary Figure S1A**). uvsX is bound to the primer by its coenzyme uvsY, and continuously binds to and separates from the primer, resulting in continuous amplification, a dynamic cyclic process mediated by ATP. In the presence of ATP, uvsX binds the primer in concert with ATP. Following ATP hydrolysis, gp32 binds the primer instead of uvsX, and gp32 detaches from the primer in the presence of uvsY and uvsX rebinds the primer, maintaining a dynamic equilibrium between uvsX, uvsY, and gp32 (**Supplementary Figure S1B**).

RPA reactions were performed using the TwistAmp^®^ Liquid DNA Amplification Kit (TwistDx Inc., Maidenhead, UK) according to the manufacturer’s instructions. A 50 µL reaction contained 25 μL of 2× reaction buffer, 5 μL of 10× Basic e-mix, 2.5 μL of 20× core mix, 2.4 μL of 10 μM forward primer, 2.4 μL of 10 μM reverse primer, and 9.2 μL of distilled water. 2.5 µL of 280 mM magnesium acetate and 1 μL of the template were added to the lid of the reaction tube. After brief centrifugation, the reaction mixture was incubated at 37°C for 30 min. The RPA amplification products were purified using a PCR Cleaning Kit (Shanghai Meiji Biotechnology Co., Ltd., Shanghai, China) and electrophoresed on a 2% agarose gel.

### RPA-LFS With Primers and a Probe

The reverse primers and probes were modified at the 5′ end with biotin and fluorescein isothiocyanate (FITC) (Sangon Biotech (Shanghai) Co., Ltd., Shanghai, China). The Nfo enzyme in the reaction system recognizes and cleaves the purine-free and pyrimidine-free [THF] site, and because of the strand replacement activity of Bsu polymerase, the DNA strand after the [THF] site is replaced, and amplification is initiated (Cossio et al., 2021). RPA reactions were set up using the TwistAmp^®^ DNA Amplification info Kit (TwistDx). The reaction mixture consisted of 29.5 μL of rehydration buffer, 2.1 μL of 10 μM forward primer, 2.1 μL of 10 μM reverse primer, 0.6 μL of 10 μM probe, and 12.2 μL of distilled water. To initiate the reaction, 1 μL of the template and 2.5 μL of 280 mM magnesium acetate were added into the mixture. After brief centrifugation, the reaction mixture was incubated at 30–45°C for 5–35 min.

Because of the very high sensitivity of colloidal gold test strips, only a small amount of product is needed for detection, and an appropriate dilution is required when the product concentration is too high. When the diluted product is added dropwise to the sample pad, both ends of the amplification product are labeled with biotin and FITC. FITC binds AuNPs, and, when passing through the detection line of streptavidin, biotin binds streptavidin, and the other end passes through gold nanoparticles (AuNPs) to show a positive signal (**Supplementary Figure S1C**). A total of 2 µL of the amplification products were used for LFS detection (Ustar Biotechnologies Ltd., Hangzhou, China). The amplification products were added to the sample pad of LFS, and the stick of LFS was inserted into 100 μL of the sample buffer (Ustar Biotech) for 2 min followed by visual reading.

### Detection Limit of RPA–LFS Technology

Nucleic acid was extracted from sterile cerebrospinal fluid and mixed with the *C. neoformans* genome to observe whether the components of sterile cerebrospinal fluid would interfere with the RPA reaction. To determine whether contamination of other strains would interfere with detection sensitivity, 10^5^ CFU/μL or 1 ng/μL of heat-treated *Candida albicans* culture or genomic DNA was added to 10-fold dilutions of heat-treated *C. neoformans* culture (10^5^–10^10^ CFU/μL) or genomic DNA (1 ng/μL–10 fg/μL).

### Evaluation of the Application of RPA-LFS Technology in Clinical Specimen Examination

RPA-LFS technology was used in clinical specimen examination. At the same time, the Indian ink staining method, which is widely used clinically, was used for parallel detection of clinical specimens. A total of 487 cerebrospinal fluids obtained clinically were examined. Finally, all samples were tested by conventional incubation. The Chi-square (x2) test was used to calculate differences among two assays testing the same clinical sample. Statistical analysis was conducted using SPSS software. P < 0.05 or P < 0.01 were both considered significant.

## Results

### Design and Screening of Primer-Probe Sets for the RPA-LFS System

**Figure 1:**
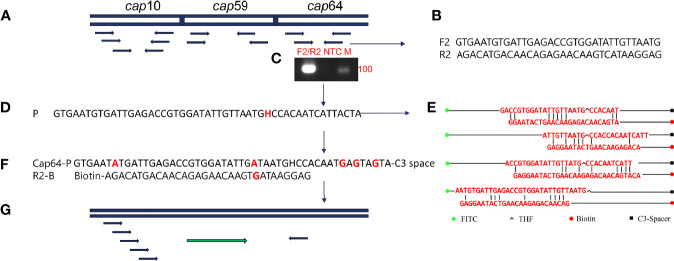
Primer and probe design screening strategy. **(A)** Detection of target gene selection. **(B)** Sequence information of F2/R2. **(C)** Agarose gel showing PCR amplification using primers F2/R2 and *C neoformans* genomic DNA as the template. **(D)** Sequence information of the candidate probe P. **(E)** Probe and reverse primers form the cross dimer. **(F)** Modified probe and reverse primer sequence information. **(G)** Schematic diagram of forward primer screening.

**Figure 2:**
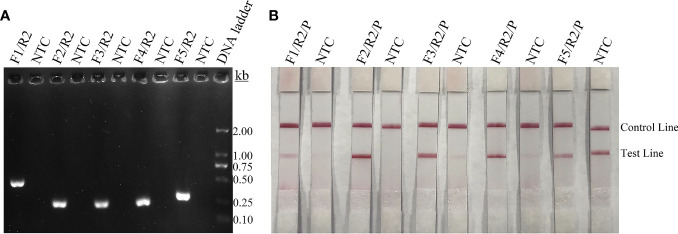
Screening of primers and probes. **(A)** Agarose gel showing PCR amplification of the primers using *C neoformans* genomic DNA as the template. The primer pair name is indicated at the top of each lane. NTC, no-template control of the respective primer pair. The band sizes of the DNA ladder are shown on the right. **(B)** Lateral flow strip (LFS) results of recombinase polymerase amplification (RPA) with different primer-probe sets. The name of each primer-probe set is indicated at the top of each strip. NTC, no-template control. The positions of test and control lines are marked on the right. The template was *C. neoformans* genomic DNA and reactions were performed at 37°C for 20 min.

**Figure 3:**
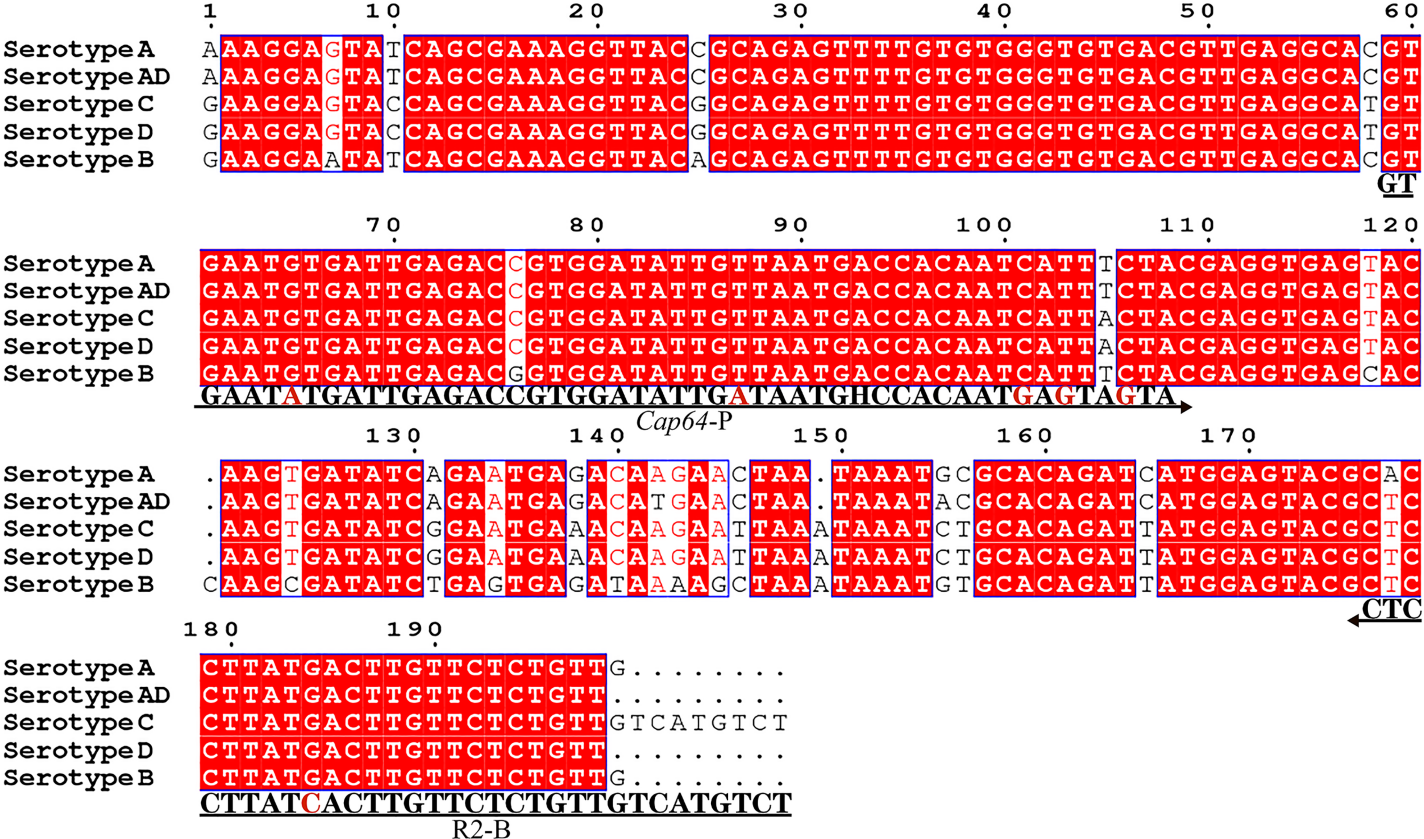
Targeting fragment of the primer-probe set R2/P. Alignment of the targeted DNA fragments from five different serotypes of *C. neoformans*/*C. gattii* was performed by NCBI BLAST. The serotypes are indicated at the beginning of each sequence. The sequences corresponding to the primers and the probe are written under their positions in the alignment. The arrow lines indicate the direction of extension of the primer and probe. The tetrahydrofuran (THF) site is represented by a “H”.

The capsular-related genes *CAP10*, *CAP59*, and *CAP64* from the *C. neoformans genome* were selected as the detection target for RPA-LFS. The NCBI Primer-BLAST search for primer candidates on the sequence of genes *CAP10*, *CAP59*, and *CAP*64 returned seven potential primer pairs (**Supplementary Table S2**). These primers were tentatively screened by amplification of the target gene fragment with the no-template control. The amplification products were electrophoresed on an agarose gel to compare the amplification performance of the target and primer-dimer formation in the no-template control. The primer pair showing the best amplification performance without a sign of cross-dimer formation was selected (**Figures 1A** **–C**). Candidate probes were obtained by extending the forward primer F2 by 16 bp at the 3′ end. All possible cross-dimers generated by this probe and the reverse primer were predicted, and subsequently, the bases were modified until no dimers could be formed (**Figures 1D** **–F**). Finally, five forward primers upstream of the probe were designed, screened, and tested (**Figure 1G**). PCR amplification and gel electrophoresis showed that all five primer pairs could effectively amplify the target gene *CAP64* (**Figure 2A**) and RPA-LFS results showed that only F2/R2/P met the detection requirements (**Figure 2B**). The sequence comparison of the different serotypes indicated that the primer-probe combination F2/R2/P could detect different serotypes of *C. neoformans/C. gattii* (**Figure 3**). Thus, F2/R2/P was used in subsequent experiments.

### Optimization of RPA-LFS Conditions

To optimize the reaction temperature of the RPA-LFS system, the RPA assay was performed at temperatures ranging from 35 to 45°C. The reaction time was set at 30 min, and the amplification results were analyzed by LFS. The pink band at the Test Line was visible at all temperatures and most visible at 37°C (**Supplementary Figure S2A**). Furthermore, the RPA reaction time was screened from 5 to 35 min. The pink band at the Test Line appeared at 10 min and became darker from 15 min. After 20 min, the darkness of the band did not change significantly (**Supplementary Figure S2B**). Thus, 37°C and 20 min were selected as the optimal reaction temperature and time for RPA.

### Detection Limit of the RPA-LFS System for *C. neoformans*

To determine the detection limit of the RPA-LFS system for *C. neoformans*, a 10-fold series dilution of the *C. neoformans* genome ranging from 10^0^ to 10^5^ CFU/µL was tested (reaction volume: 50 µL, 1 µL *C. neoformans* genome was added to each reaction). Although weak, a pink band still appeared at the Test Line with 10 CFU/µL. Also, the pink band darkened with the increasing concentrations of *C. neoformans* (**Supplementary Figure S3A**). In a similar manner, 10-fold serial dilutions of purified *C. neoformans* genomic DNA were tested. As low as 100 fg of *C. neoformans* genomic DNA could be detected (**Supplementary Figure S3C**). To test if the system could resist the interference of other fungal DNA, 10^5^ CFU/µL or 1 ng of the genomic DNA of *C. albicans*, species member of the fungal microbiome, was added into the RPA reactions along with the dilutions of heat-treated *C. neoformans* culture or genomic DNA. The heat-treated *C. albicans* culture or genomic DNA did not interfere with the detection of *C. neoformans* (**Supplementary Figure S3B, D**). We concluded that the detection limit of the RPA-LFS system was 10 CFU per reaction with DNA purification or 1 pg of genomic DNA/50 µL. The detection sensitivity was not affected by the presence of other fungal DNA.

### Detection Specificity of the RPA-LFS System

**Figure 4:**
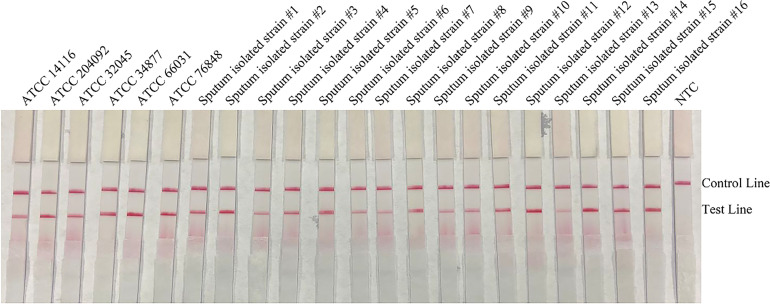
Detection inclusivity among different reference strains and sputum isolate strains. LFS results of RPA amplification of different genomic DNA templates. The names of the strains are indicated on top of each strip. NTC, no-template control. The positions of the Control and Test lines are indicated on the right of the image. The reactions were performed at 37°C for 20 min.

**Figure 5:**
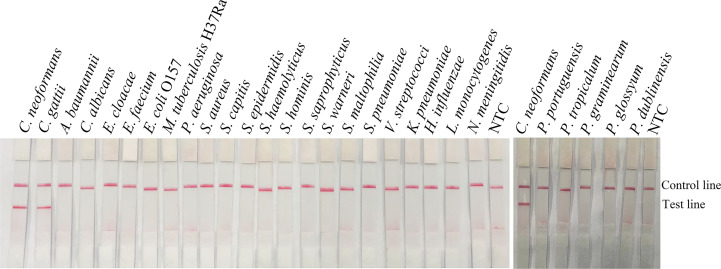
Detection specificity among common pathogens. LFS results of RPA of different bacterial culture templates. The species of bacteria are indicated at the top of each strip. NTC, no-template control. The positions of the Control and Test lines are indicated on the right of the image. The reactions were performed at 37°C for 20 min.

To confirm the inclusivity and specificity of the primer-probe set, the primer pair was tested for RPA-LFS amplification of 6 reference strains, 16 clinical isolates strains, and other pathogenic bacterial species. Since *C. gattii* is a newly discovered strain, apart from the traditional culture method and the Indian ink staining method, no method has been found to distinguish *C. neoformans* and *C. gattii*. Six reference strains and 16 clinical isolates strains of *C. neoformans* and *C. gattii* showed a positive result (**Figure 4**), and the other bacterial and fungal cultures were negative (**Figure 5**), indicating that the primer-probe set showed good inclusivity and specificity toward *C. neoformans*/*C. gattii* and would not likely cross-react with other pathogenic bacteria and fungi. All non-pathogenic *C. neoformans* species were negative in the test (data not shown), suggesting that the system could detect *C. neoformans*/*C. gattii* but not non-pathogenic strains.

### Application of the RPA-LFS System for *C. neoformans*/*C. gattii* Detection

The RPA-LFS system was applied to detect *C. neoformans*/*C. gattii* in the 487 cerebrospinal fluid samples, and the performance was compared with the India ink staining method. All 90 positive samples were successfully detected by the RPA-LFS system. At the same time, India ink staining method detected only 78 positive samples; 12 samples were missed. All samples were morphologically observed using conventional culture methods, and the assay and RPA-LFS results were consistent. Therefore, the detection performance of RPA-LFS was significantly better than that of the India ink staining method (**Table 1**). No false-positive results were observed for either assay.

**Table 1:** Determination of coincidence rate between the RPA-LFS assay and India ink staining methods in clinical samples.

RPA-LFS assay					
		Positive	Negative	Total	X^2^
India ink staining methods	Positive	78	0	78	
					10.08
	Negative	12	397	409	P<0.05
Total		90	397	487	

## Discussion

*C. neoformans* is an opportunistic pathogen widely distributed in nature that causes mild inflammation in the lungs and is found mostly in the respiratory tract, but also in broken skin and the intestinal tract (Duperval et al., 1977; Cogliati et al., 2016). Thus, there is an urgent clinical need for a rapid, sensitive, and specific assay capable of rapidly detecting these fungi at an early stage of disease progression. With the rapid development of molecular diagnostic techniques, RPA, a promising isothermal amplification technique that can be combined with flow metric chromatography strips, is increasingly being used for field detection of pathogenic bacteria (Wu et al., 2017; Tian et al., 2018). Yang et al. (2021) used RPA-LFS to detect *P. aeruginosa*, for which the amplification process can be completed within 30 min at a constant temperature of 37°C, and the results are visible to the naked eye on the LFS within 10 min. The assay shows high sensitivity with a detection limit of 3.05 CFU/reaction. Jiang et al. (2020) developed a sensitive and rapid recombinase polymerase amplification assay for the detection of phagocytosed anaerobes that can reliably detect 125 bacteria/mL in human blood using genomic DNA from humans or DNA from other closely related pathogens such as *Ehrlichia flatulentis*, *Ehrlichia* spp. such as *Ehrlichia flatus*, *Ehrlichia chaffeensis*, *Orientia tsutsugamushi* and *Rickettsia rickettsii* without nonspecific amplification, demonstrating a high level of specificity. Ayfan et al. (2021) developed a rapid and simple molecular test to combine recombinase polymerase amplification and lateral flow assays to detect bla NDM-type and bla VIM-type carbapenemase genes. When using DNA extracts, the test provided results in approximately 15 min, with a detection limit of 9.2 copies/μL for the bla NDM-type assay and 7.5 copies/μL for the bla VIM-type assay analysis, and successfully detected all 57 strains with the carbapenemase-encoding gene. Ma et al. (2019) developed a lateral flow recombinase polymerase amplification assay for rapid and visual detection of *C. neoformans*/*C. gatti* in cerebrospinal fluid. The resulting LF-RPA assay detected 0.64 pg of *C. neoformans* genomic DNA per reaction in 10 min and was highly specific for Cryptococcus. The system functioned over a wide temperature range of 25 to 45°C. The overall sensitivity and specificity were 95.2% and 95.8%, respectively. As an amplification template for LF-RPA analysis, both cell lysates and genomic DNA yielded similar experimental results. In this study, our RPA-LFS system delivered results within 20 min at an isothermal temperature of 37°C with a lower limit of detection of 10 CFU/μL or 1 fg/μL.

With increasing popularity of the RPA-LFS technique, this method is being used for the detection of pathogenic bacteria due to its high specificity, high sensitivity, and reduced need for specialized equipment (Tian et al., 2018). Compared with India ink staining, this method has a high detection rate and no false negative results, indicating that the RPA-LFS assay is superior, allowing accurate feedback of test results and competing for valuable time for the treatment of critically ill patients (Paes et al., 2019). Immunochromatographic test strips based on antigen-antibody combinations are also being developed, and unlike molecular diagnostic-based RPA reactions, which require a certain period of disease progression to enrich for antigenic molecules that can be used for detection, are inferior to RPA reactions early in the disease process to detect nucleic acid material released by pathogenic bacteria as early as possible (Min et al., 2020). As in the case of the new coronavirus test, molecular diagnostics are being used more often than ELISA to screen large numbers of positive patients in the early stages of disease progression. Many investigators have also reported relatively low consumption of RPA-LFS in terms of reagents used, and as capacity continues to increase, the RPA-LFS testing system is expected to be more cost effective (Jarvis et al., 2011). However, RPA-LFS, like all current assays, has some shortcomings, including false positives due to aerosol contamination, which can be addressed by strict compartmentalization, i.e., separate spaces for reagent preparation, sample addition, and open-cap detection, or with the use of special aerosol contamination prevention devices. In addition, since the RPA reaction requires the participation of multiple enzymes, the storage conditions for the reagents are demanding (Wang et al., 2019; Ayfan et al., 2021).

The RNA-LFS system for detecting *C. neoformans*/*C. gattii* developed in this study focused on an improved primer-probe screening strategy compared to Ma’s method to avoid false-positive results by reducing cross dimer generation through appropriate base mismatches (Ma et al., 2019). We designed multiple pairs of forward and reverse primers on target gene and obtained specific primer pairs by RPA amplification (Fischer et al., 2016). Approximately 15 bases are pulled-down after the upstream primers as probes, and the possible formation of cross dimers by the probe and downstream primers was predicted by Primer Premier 5.0 and base substitution was performed until all possible cross dimers were eliminated (Oni et al., 2018). Subsequently, multiple upstream primers were designed upstream of the probe, and the best primer-probe combination was obtained by combining different upstream primers with downstream primers two by two (Piepenburg et al., 2006; Wang et al., 2019). During base modification, care should be taken to avoid introducing mismatches at the 3´ end of the primers or probes, as this tended to degrade their amplification performance (Deng et al., 2020). Although RPA reactions can tolerate seven base mismatches, fewer base substitutions can maximize the detection performance of the established RPA-LFS system (Liu et al., 2019b). The pretreatment of sterile cerebrospinal fluid samples is also a problem in the detection of *C. neoformans* by RPA-LFS. *C. neoformans*/*C. gattii* is a yeast-like fungus with a thick pod membrane that makes it difficult to release its genomic DNA, and it is not easy to release the genetic DNA for detection by the usual boiling method. The method in our study saves the detection time of this detection system and meets the rapid, sensitive field test requirements.

## Conclusion

The novel clinical rapid visualization molecular diagnostic technique for *C. neoformans*/*C. gattii* based on recombinant enzyme polymerase isothermal amplification combined with colloidal gold test strips (RPA-LFS) developed in this study could obtain test results within 20 min, and had high specificity, high sensitivity, low instrument dependence, did not require professional trained laboratory personnel, and was highly operable. It can be used in the field to meet the needs of bedside diagnostics or the needs of remote hospitals with weak conditions, and is of great importance for the rapid detection of *C. neoformans*/*C. gattii*.

## Data Availability Statement

The original contributions presented in the study are included in the article/**Supplementary Material**. Further inquiries can be directed to the corresponding authors.

## Ethics Statement

The studies involving human participants were reviewed and approved by the Medical Ethics Committee of the Second People’s Hospital of Lianyungang City. The patients/participants provided their written informed consent to participate in the study.

## Author Contributions

KW, WGZ, and WL conceived and designed the experiments. LW, FW, MDZ, XZG, PZ, YZL, and YW performed the experiments. HMC, NL, and QZ collected the clinical strains. LPL, WJZ, and XL analyzed the data. LW and YJC wrote the manuscript. All authors reviewed, revised, and approved the final report. All authors contributed to the article and approved the submitted version.

## Funding

This study was supported by grants from the 2021 Lianyungang Sixth “521 Project” scientific research funding projects(LYG06521202160), the Natural Science Foundation of Jiangsu Province (grant number BK20191210), the “Project 333” training fund of Jiangsu Province (grant number BRA2019248), the Jiangsu University Clinical Medicine Science and Technology Development Fund Project (grant number JLY2021088), the Lianyungang City Health Science and Technology Project (grant number 202122), the China Agriculture Research System of MOF and MARA (grant number CARS-18-ZJ0207), and the National Key R and D Program of China, key projects of international scientific and technological innovation cooperation (grant number 2021YFE0111100).

## Conflict of Interest

The authors declare that the research was conducted in the absence of any commercial or financial relationships that could be construed as a potential conflict of interest.

## Publisher’s Note

All claims expressed in this article are solely those of the authors and do not necessarily represent those of their affiliated organizations, or those of the publisher, the editors and the reviewers. Any product that may be evaluated in this article, or claim that may be made by its manufacturer, is not guaranteed or endorsed by the publisher.

## Supplementary Material

Supplementary Figure 1 Schematic diagram of RPA-LFS. **(A)** Schematic representation of the RPA working. The uvsX is continuously bound to the primer, and continuously binds to and separates from the primer, resulting in a continuous amplification reaction. **(B)** Schematic representation of the dynamic cyclic process mediated by ATP working. uvsX binds the primer in concert with ATP. After ATP hydrolysis, gp32 binds the primer instead of uvsX, and gp32 detaches from the primer in the presence of uvsY and uvsX rebinds the primer. **(C)** Schematic representation of the lateral flow strip (LFS) working. The diluted product is added dropwise to the sample pad, both ends of the amplification product are labeled with biotin and FITC, respectively. FITC binds to AuNPs, and when passing through the detection line of streptavidin, biotin binds to streptavidin, and the other end passes through gold nanoparticles (AuNPs) to show a positive signal.

Supplementary Figure 2 Optimal reaction temperature and time of the RPA-LFS system. **(A)** LFS results of RPA amplifications under different temperatures. The temperatures under which the RPA reactions were performed are indicated at the top of each strip. The amplification template was *C. neoformans* genomic DNA. NTC, no-template control performed at 40°C. **(B)** LFS results of RPA amplifications with different time lengths, which are indicated at the top of each strip. The amplification template was *C. neoformans* genomic DNA. NTC, no-template control performed for 25 min. The positions of the Control and Test lines are indicated on the right of the images.

Supplementary Figure 3 Detection limit of the RPA-LFS system. **(A, B)** LFS results of RPA amplifications with different amounts of *C. neoformans* culture. The amounts (in CFU) added to the RPA reactions are indicated at the top of each strip. In **(B)**, 10^5^ CFU/μL of the culture of *C*. *neoformans* was added to the reactions in addition to the *C*. *neoformans* culture. **(C, D)** LFS results of RPA amplifications with different amounts of *C. neoformans* genomic DNA. The amounts added to the RPA reactions are indicated at the top of each strip. In **(D)**, 1 ng of the genomic DNA of *C. albicans* was added to the reactions in addition to the *C. neoformans* genomic DNA. NTC, no-template control. The reactions were performed at 37°C for 20 min. The positions of the Control and Test lines are indicated on the right of the images.
