# Supplementary figures and images for: Development and Application of Rapid Clinical Visualization Molecular Diagnostic Technology for Cryptococcus neoformans/C. gattii Based on Recombinase Polymerase Amplification Combined With a Lateral Flow Strip
Source: Front Cell Infect Microbiol. 2022 Jan 12;11:803798. doi: 10.3389/fcimb.2021.803798 (PMC8790172; doi:10.3389/fcimb.2021.803798)

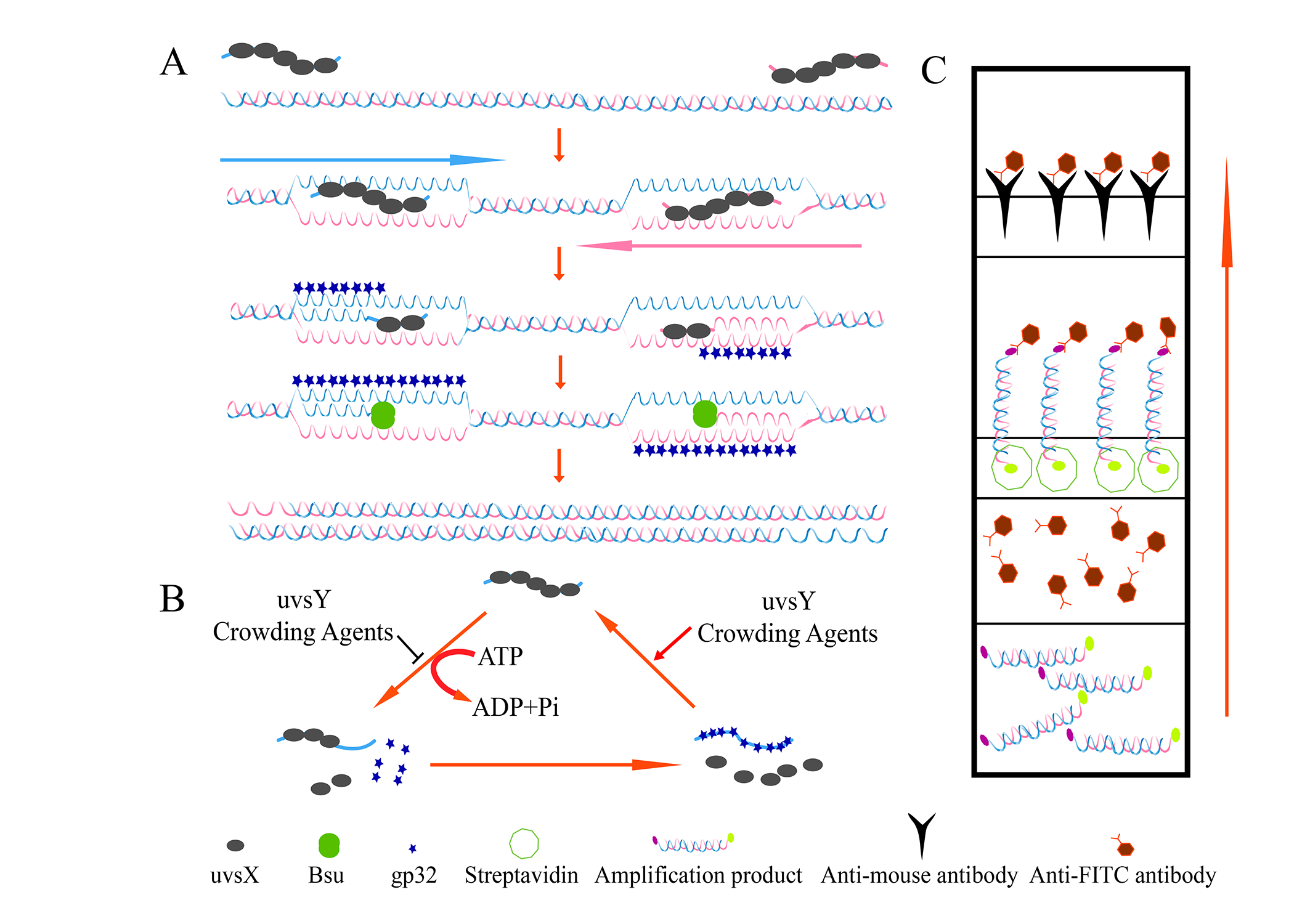

Supplement: Supplementary Figure 1 — Schematic diagram of RPA-LFS. (A) Schematic representation of the RPA working. The uvsX is continuously bound to the primer, and continuously binds to and separates from the primer, resulting in a continuous amplification reaction. (B) Schematic representation of the dynamic cyclic process mediated by ATP working. uvsX binds the primer in concert with ATP. After ATP hydrolysis, gp32 binds the primer instead of uvsX, and gp32 detaches from the primer in the presence of uvsY and uvsX rebinds the primer. (C) Schematic representation of the lateral flow strip (LFS) working. The diluted product is added dropwise to the sample pad, both ends of the amplification product are labeled with biotin and FITC, respectively. FITC binds to AuNPs, and when passing through the detection line of streptavidin, biotin binds to streptavidin, and the other end passes through gold nanoparticles (AuNPs) to show a positive signal. [file Image_1.tif]

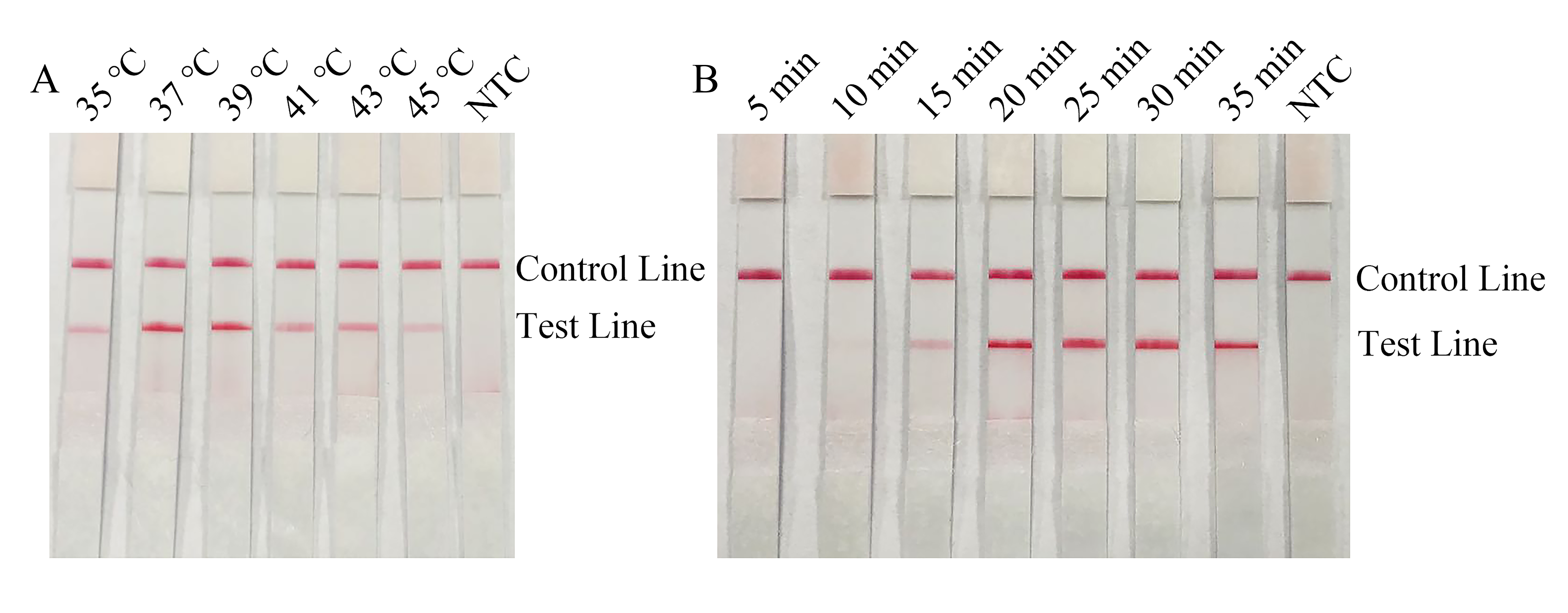

Supplement: Supplementary Figure 2 — Optimal reaction temperature and time of the RPA-LFS system. (A) LFS results of RPA amplifications under different temperatures. The temperatures under which the RPA reactions were performed are indicated at the top of each strip. The amplification template was C. neoformans genomic DNA. NTC, no-template control performed at 40°C. (B) LFS results of RPA amplifications with different time lengths, which are indicated at the top of each strip. The amplification template was C. neoformans genomic DNA. NTC, no-template control performed for 25 min. The positions of the Control and Test lines are indicated on the right of the images. [file Image_2.tif]

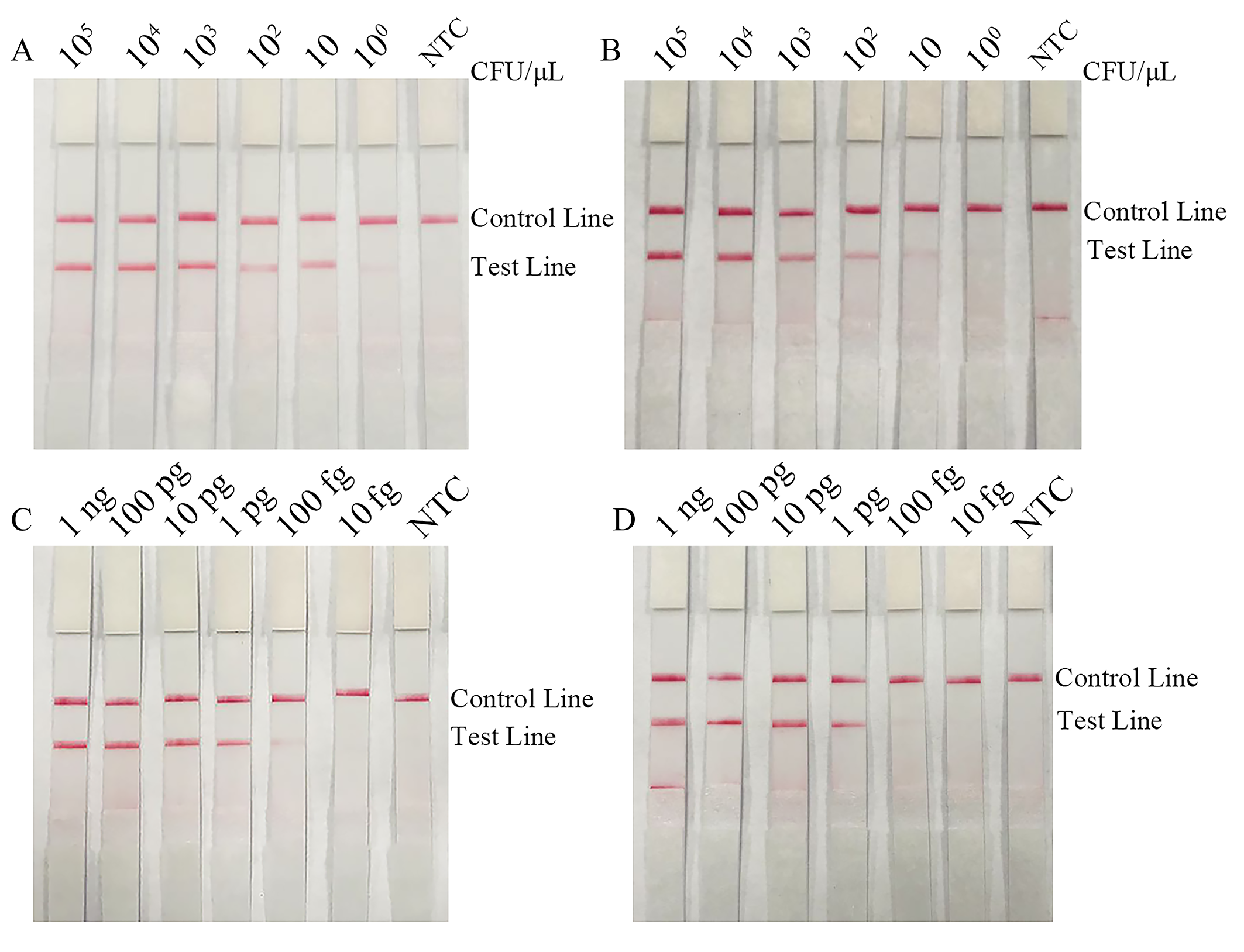

Supplement: Supplementary Figure 3 — Detection limit of the RPA-LFS system. (A, B) LFS results of RPA amplifications with different amounts of C. neoformans culture. The amounts (in CFU) added to the RPA reactions are indicated at the top of each strip. In (B), 105 CFU/μL of the culture of C. neoformans was added to the reactions in addition to the C. neoformans culture. (C, D) LFS results of RPA amplifications with different amounts of C. neoformans genomic DNA. The amounts added to the RPA reactions are indicated at the top of each strip. In (D), 1 ng of the genomic DNA of C. albicans was added to the reactions in addition to the C. neoformans genomic DNA. NTC, no-template control. The reactions were performed at 37°C for 20 min. The positions of the Control and Test lines are indicated on the right of the images. [file Image_3.tif]
