# Supplementary material for: Development and Application of Rapid Clinical Visualization Molecular Diagnostic Technology for Cryptococcus neoformans/C. gattii Based on Recombinase Polymerase Amplification Combined With a Lateral Flow Strip
Source: Front Cell Infect Microbiol. 2022 Jan 12;11:803798. doi: 10.3389/fcimb.2021.803798 (PMC8790172; doi:10.3389/fcimb.2021.803798)
Supplement: Supplementary file 4 [file Table_1.docx]

**Supplementary materials**

Supplementary Table S1. Bacterial strains used in the study

| Species | **Source** | **Strain Amount** | **Origin/Designation** | RPA-LFS | **India ink staining methods** | **Culture-biochemical Methods** |
| --- | --- | --- | --- | --- | --- | --- |
| *Cryptococcus neoformans* | Reference strain | 1 | ATCC 14116 | Positive | Positive | Positive |
| *C. neoformans* | Reference strain | 1 | ATCC 204092 | Positive | Positive | Positive |
| *C. neoformans* | Reference strain | 1 | ATCC 32045 | Positive | Positive | Positive |
| *C. neoformans* | Reference strain | 1 | ATCC 34877 | Positive | Positive | Positive |
| *C. neoformans* | Reference strain | 1 | ATCC 66031 | Positive | Positive | Positive |
| *C. neoformans* | Reference strain | 1 | ATCC 76848 | Positive | Positive | Positive |
| *C. neoformans* | Sputum isolated strain | 16 | Lianyungang, China | Positive | Positive | Positive |
| *Cryptococcus gattii* | Reference strain | 1 | ATCC 34877 | Positive | Positive | Positive |
| *Acinetobacter baumannii* | Sputum isolated strain | 1 | Lianyungang, China | Negative | Negative | Negative |
| *Candida albicans* | Reference strain | 1 | ATCC 10231 | Negative | Negative | Negative |
| *Enterobacter cloacae,* | Sputum isolated strain | 1 | Lianyungang, China | Negative | Negative | Negative |
| *Enterococcus faecium* | Sputum isolated strain | 1 | Lianyungang, China | Negative | Negative | Negative |
| *Escherichia coli O157* | Sputum isolated strain | 1 | Lianyungang, China | Negative | Negative | Negative |
| *Mycobacterium tuberculosis H37Ra* | Sputum isolated strain | 1 | Lianyungang, China | Negative | Negative | Negative |
| *Pseudomonas aeruginosa* | Sputum isolated strain | 1 | Lianyungang, China | Negative | Negative | Negative |
| *Staphylococcus aureus* | Sputum isolated strain | 1 | Lianyungang, China | Negative | Negative | Negative |
| *Staphylococcus capitis* | Sputum isolated strain | 1 | Lianyungang, China | Negative | Negative | Negative |
| *Staphylococcus epidermidis* | Sputum isolated strain | 1 | Lianyungang, China | Negative | Negative | Negative |
| *Staphylococcus haemolyticus* | Sputum isolated strain | 1 | Lianyungang, China | Negative | Negative | Negative |
| *Staphylococcus hominis* | Sputum isolated strain | 1 | Lianyungang, China | Negative | Negative | Negative |
| *Staphylococcus saprophytics* | Sputum isolated strain | 1 | Lianyungang, China | Negative | Negative | Negative |
| *Staphylococcus wameri* | Sputum isolated strain | 1 | Lianyungang, China | Negative | Negative | Negative |
| *Stenotrophomonas maltophilia* | Sputum isolated strain | 1 | Lianyungang, China | Negative | Negative | Negative |
| *Streptococcus pneumonia* | Sputum isolated strain | 1 | Lianyungang, China | Negative | Negative | Negative |
| *Viridans streptococci* | Sputum isolated strain | 1 | Lianyungang, China | Negative | Negative | Negative |
| *Klebsiella pneumoniae* | Sputum isolated strain | 1 | Lianyungang, China | Negative | Negative | Negative |
| *Haemophilus influenzae* | Sputum isolated strain | 1 | Lianyungang, China | Negative | Negative | Negative |
| *Listeria monocytogenes* | Sputum isolated strain | 1 | Lianyungang, China | Negative | Negative | Negative |
| *Neisseria meningitidis* | Sputum isolated strain | 1 | Lianyungang, China | Negative | Negative | Negative |
| *Pseudostelium portuguensis* | Sputum isolated strain | 1 | Lianyungang, China | Negative | Negative | Negative |
| *Pseudostelium tropicalum* | Sputum isolated strain | 1 | Lianyungang, China | Negative | Negative | Negative |
| *Pseudomonas graminearum* | Sputum isolated strain | 1 | Lianyungang, China | Negative | Negative | Negative |
| *Pseudostelium glossyum* | Sputum isolated strain | 1 | Lianyungang, China | Negative | Negative | Negative |
| *Pseudomonas dublinensis* | Sputum isolated strain | 1 | Lianyungang, China | Negative | Negative | Negative |

ATCC, American Type Culture Collection, Rockville, MD, USA.

Supplementary Table S2. Design and screening of primer-probe sets for the RPA-LFS system

| Primer pair | Primer sequences | Primer size |
| --- | --- | --- |
| *CAP*10-F1 | GACTATCCATGCTCTTTACCCCATGTTCTC | 30 |
| *CAP*10-R1 | AAATAGTAGTGACTGGGAATAAGAATGTCGCTG | 33 |
| *CAP*10-F2 | CTGAACAAAGATATGATGGATGTGGCGTTC | 30 |
| *CAP*10-R2 | AAATACTTGTGTCTCCAGTTCTCACCAAGAG | 31 |
| *CAP*10-F3 | CTCTGACTCTTCCAATGTCAACAAAACTGTC | 31 |
| *CAP*10-R3 | CTTTGTGAACGCCACATCCATCATATCTTT | 30 |
| *CAP*59-F1 | TAAAAAGATATGTGTGCGTGTATGGGGGTG | 30 |
| *CAP*59-R1 | AGGCCATAAGATTCAGTCAAATGTAATAATCGGAA | 35 |
| *CAP*59-F2 | AAGCATTACCGAAATATGCCTGGATGGTAGA | 31 |
| *CAP*59-R2 | TTCAGTCAAATGTAATAATCGGAACCCCAACTC | 33 |
| *CAP*64-F1 | TAATCTCAAGTTATTTCTGTCCATCTCTCCACATC | 35 |
| *CAP*64-R1 | TTCTGGGGTATATGAAAGTAGTGAAGGTAAACAAG | 35 |
| *CAP*64-F2 | GTGAATGTGATTGAGACCGTGGATATTGTTAATG | 34 |
| *CAP*64-R2 | AGACATGACAACAGAGAACAAGTCATAAGGAG | 32 |
| F1 | TTACGCCACTCCCAAGAACGCCTCTGAATACCC | 33 |
| F2 | ACGCCACTCCCAAGAACGCCTCTGAATACCCTG | 33 |
| F3 | CGGAGAAGGAGTACCAGCGAAAGGTTACGGCAGAG | 35 |
| F4 | GAGAAGGAGTACCAGCGAAAGGTTACGGCAGAG | 33 |
| F5 | GAAGGAGTACCAGCGAAAGGTTACGGCAGAGTTT | 34 |
| *CAP*64-P | FITC-GTGAATATGATTGAGACCGTGGATATTGATAATG[THF]CCACAATGAGTAGTA-/C3-spacer/ | 46 |
| R2-B | Biotin-AGACATGACAACAGAGAACAAGTGATAAGGAG | 32 |
